# Domestic Summary

**Published:** 1839-03-16

**Authors:** 


### DOMESTIC SUMMARY.

*The State Lunatic Hospital at Worcester.*—The following are selected from the large number of interesting facts contained in Dr. Woodward's last annual report.

In the course of the last year, there have been admitted 177 patients, a greater number than has heretofore been admitted in any single year. Of these, 96 were males and 81 were females; 82 were of less duration than one year, 45 males and 37 females, and 95 of longer duration than one year, 51 males and 44 females.

At the close of the year there were in the hospital 218 patients, of whom 115 were males and 103 were females. Of this number of cases 82 are of duration less than one year, and 190 of duration longer than one year.

During the year there have been in the hospital 362 patients, 177 of whom were admitted in the course of the year, and 185 were in the hospital at the commencement of the year.

There have been discharged during the year, including deaths, 144 patients, of whom 84 were males and 60 were females. 76 of these recovered, 45 males and 31 females; 24 were improved, 11 males and 13 females; 14 were not improved, 8 males and 6 females; 14 were discharged harmless and incurable, for want of room, 10 males and 4 females; and 16 have died, 10 males and 6 females.

Of this number of cases discharged, 74 were of less duration than one year, 47 males and 27 females. Of these 64 recovered, 38 males and 26 females; 6 were discharged improved, 2 males and 4 females; 4 have died, 3 males and 1 female.

Of the number of cases discharged, 70 were of duration longer than one year. Of these 42 were males and 28 females; 12 recovered, 7 males and 5 females; 18 were discharged improved, 10 males and 8 females; and 12 died, 7 males and 5 females.

Respecting the ages of patients now in the hospital, of any 10 years, the greatest number of patients are between 30 and 40 years of age, few are under 20, and more are between the ages of 40 and 50 than between 20 and 30. This, it is believed, is different from the fact with most hospitals for the insane, and may be accounted for, in part, from the accumulation of old cases in this hospital, which was originally designed principally for incurables, many of whom will continue within its wards while life remains.

The average number of the year 1837 was 163; the average number for the year 1838 is 211, a difference of 55 in the average of the two years.

At this time the hospital is as full of patients as it is desirable that it should ever be, and without the lodges, which should never be estimated as a part of the accommodations of the establishment, is already more than full.

The recoveries of mania are about 60 per cent., and the recoveries of melancholia about 59 per cent., while recoveries of dementia, as we use the term, are from 2 to 3 per cent. only.

The great list of employments in the table show conclusively that all mankind, of whatever pursuits, are liable to the evil, and that little can be said of the occupation as a cause of the insanity in any case.

The number of single persons continues to be much larger than the married, as has always been the case in the hospital. During the last year we have received 101 patients that have never been married, 65 married, and 11 in a state of widowhood.

Intemperance continues to be a prominent cause, but we are happy to think it is less frequent than formerly. It will elsewhere be recorded that this cause, during the first three years of the hospital, gave origin to 25 per cent. of the cases of insanity admitted, while it is supposed to be the cause in but 14 per cent. of the cases admitted the last three years. If this is any indication of the proportionate diminution of its influence in other respects, unfavourable to public health and public morals, the prospect is most cheering. We have had no case of delirium tremens for the last year, and very few since the institution was opened.

The number of admissions from religious causes has been about the same as usual the past year. A subject so deeply interesting to the human mind as its eternal well-being, must ever have an agency in the production of insanity; these cases come in bold relief before us, and we deprecate the influence which has produced them. All the most valuable institutions of society, however, are liable to the same objections—marriage, education and civilization, as well as Christianity, are the causes of insanity in many cases, though it is not the legitimate tendency of any of them to produce this effect.

The number of admissions from masturbation, the last year, has been less, and the cases of a more favourable character. 6 cases only are known to have arisen from this cause; but probably 3 or 4 others may have done so. 4 or 5 of these cases have recovered, and have been discharged with such feelings of the nature and tendency of the practice, as it may confidently be hoped will ensure them from future indulgence and its consequences.

The ascertained causes of insanity in the 855 cases at this hospital, rank thus: 1, intemperance; 2, ill health of all kinds; 3, masturbation; 4, domestic afflictions; 5, religious excitement; 6, loss of property and fear of poverty; 7, disappointed ambition; 8, injuries of the head; 9, use of snuff and tobacco. In a few cases, the cause of the insanity is unknown. Foreigners and citizens of other states found insane in this, have occasionally been committed, whose histories could not be ascertained. Probably we shall approximate the truth very closely in distributing the unknown causes under the above heads, according to their relative proportions.

Of the whole number of cases admitted into the hospital, 334 were of less duration than one year, of which there are recovered or supposed curable, 294, which is 88 per cent. From one to two years' duration, 118; recovered or supposed curable, 79, a fraction more than 66 per cent. From two to five years' duration, 141; recovered or supposed curable, 45, a little less than 36 per cent. From five to ten years' duration, 96; recovered or supposed curable, 12, or 12½ per cent. Over ten years' duration, 118; recovered, 4, less than 3½ per cent.

During the last year, the recoveries of patients in whom insanity commenced under 20 years of age, has been 46 per cent.; in those between 20 and 25, 51⅓ per cent.; between 25 and 30, 42 per cent.; between 30 and 35, 51 per cent.; between 35 and 40, 51 per cent.; between 40 and 45, 67 per cent.; between 50 and 55, 60 per cent.; between 55 and 60, 66 per cent.; between 60 and 65, 90 per cent.; between 65 and 70, 67 per cent.; after the age of 70, 57 per cent.

Of the deaths that have occurred in the hospital, 12 have been of recent cases, and 41 of old cases. No one has died of fever, and 4 only of inflammatory disease.

The proportion of deaths must be considered small for the number of the imbecile, feeble and diseased that have annually been brought to our care, being only 53 of 855, a little more than 6 per cent.; the average on the number in the hospital each year, is about 3½ per cent.

Recovery of insanity from certain causes: From intemperance, 51⅓ per cent. Domestic afflictions, 58 per cent. Ill health, 62½ per cent. Religious causes, 55½ per cent. Masturbation, 18½ per cent.—*Boston Medical and Surgical Jour.*

---

*A Physiological Phenomena, or the Snake man; Robert H. Copeland.*—This most singular being, perhaps, has not a parallel in medical history. He is now about 29 years old, of ordinary stature and intellect. His deformities and physical peculiarities are owing to a fright his mother received from a large rattle-snake attempting to bite her, about the sixth month of her pregnancy. For several minutes after the snake struck at her she believed herself bitten just above the ankle; and so powerfully was her mind affected, that, when she was delivered, the child's will was found to have no control over his right arm and right leg; which are smaller than his left extremities. He can use his right leg now, sufficiently to walk in a hobbling manner, but cannot retain it stationary, without the aid of the weight of his body. His right hand has the usual number of fingers, but they are smaller than those of his left hand. The wrist joint is looser than usual, and his hand stands at an angle with his arm. His front teeth are somewhat pointed, and incline backward, like the fangs of a snake. The right side of his face is sensibly affected; his mouth is drawn considerably farther on the left side: his right eye squints, has several deep grooves radiated from it, and has a very singular appearance, much resembling a snake. But perhaps the most extraordinary circumstance on record, is, that his arm, when not restrained will draw the lower part to about a right angle with the upper and sometimes two or three, but most commonly, only the fore finger will project, curved at the first joint, much resembling a snake's head and neck, when in the attitude of striking; and the whole arm will strike at an object with all the venom of a snake, and precisely in the same manner, for two or three, and some times for four or five strokes, and then the arm assumes a vibratory motion, will coil up and apply itself close against his body. During this period, his right foot and leg become excited, and if not restrained, will strike also. His face is also excited; the angle of his mouth is drawn backward, and his eye snaps more or less, in unison with the strokes of his hand, whilst his lips are always separated, exposing his teeth, which, being somewhat pointed like the fangs of a snake, causes his whole visage to assume a peculiar and snaky aspect. During infancy and childhood, the whole shape of the snake, even to its fangs, was imprinted on the anterior of his leg; but as he grew up, it became gradually obliterated, till now there is only a small depression where the snake's head was imprinted. The sight of a snake fills him with horror, and an instinctive feeling of revenge; and he is more excitable during the season of snakes; and even conversation concerning them excites him, and his arm appears more anxious to strike than when no such conversation is going on.

All the above phenomena are perfectly independent of his will, as hundreds can testify who were acquainted with him long before he had any idea of exhibiting himself publicly. This singular being was born in Carolina, and moved to Georgia in the year 1829; where he has since remained, performing such labour as he could with one hand, and by unremitting exertions has maintained his wife and an increasing family. His physical peculiarities being considered only in the light of a common deformity, he never thought of exhibiting himself publicly, till it was suggested to him by a medical friend in 1837.

We the undersigned, physicians and others, after carefully examining Mr. Robert H. Copeland, do certify, that the above description of him is substantially true. Jacob Stokes, M. D., Addison Bean, M. D., F. E. Manson, M. D., A. V. Mann, M. D., Samuel C. Elliot, M. D., of McDonough, Henry co.; Hon. Wm. Segar, M. D., of Henry county; Jas. Love, Sheriff of Henry county; Jacob Martin, Attorney at Law, Zebulon, Pike county.—*Southern Med. and Sur. Jour.*

